# A Deep Learning-Based Radiomics Model for Prediction of Survival in Glioblastoma Multiforme

**DOI:** 10.1038/s41598-017-10649-8

**Published:** 2017-09-04

**Authors:** Jiangwei Lao, Yinsheng Chen, Zhi-Cheng Li, Qihua Li, Ji Zhang, Jing Liu, Guangtao Zhai

**Affiliations:** 1 Institute of Image Communication and Network Engineering, Shanghai Jiao Tong University, Shanghai, China; 2 Department of Neurosurgery/Neuro-oncology, Sun Yat-sen University Cancer Center, State Key Laboratory of Oncology in South China, Collaborative Innovation Center for Cancer Medicine, Guangzhou, China; 3 Institute of Biomedical and Health Engineering, Shenzhen Institutes of Advanced Technology, Chinese Academy of Sciences, Shenzhen, China; 4 School of Electrical and Information Engineering, Tianjin University, Tianjin, China

## Abstract

Traditional radiomics models mainly rely on explicitly-designed handcrafted features from medical images. This paper aimed to investigate if deep features extracted via transfer learning can generate radiomics signatures for prediction of overall survival (OS) in patients with Glioblastoma Multiforme (GBM). This study comprised a discovery data set of 75 patients and an independent validation data set of 37 patients. A total of 1403 handcrafted features and 98304 deep features were extracted from preoperative multi-modality MR images. After feature selection, a six-deep-feature signature was constructed by using the least absolute shrinkage and selection operator (LASSO) Cox regression model. A radiomics nomogram was further presented by combining the signature and clinical risk factors such as age and Karnofsky Performance Score. Compared with traditional risk factors, the proposed signature achieved better performance for prediction of OS (C-index = 0.710, 95% CI: 0.588, 0.932) and significant stratification of patients into prognostically distinct groups (P < 0.001, HR = 5.128, 95% CI: 2.029, 12.960). The combined model achieved improved predictive performance (C-index = 0.739). Our study demonstrates that transfer learning-based deep features are able to generate prognostic imaging signature for OS prediction and patient stratification for GBM, indicating the potential of deep imaging feature-based biomarker in preoperative care of GBM patients.

## Introduction

Glioblastoma multiforme (GBM) is the most frequent malignant primary brain tumor in adults^1^. GBM accounts for 15% of brain tumors^2^. The median survival is only 12 to 14 months even with aggressive therapy^3^. The poor prognosis is mainly due to the spatial and temporal intra-tumor heterogeneity. This genetic heterogeneity reduces the value of invasive biopsy-based genomic analysis, but provides opportunities for medical imaging that depicts the entire tumor in a non-invasive and repeatable way. To explore the correlation between medical images and underlying genetic characteristics, radiomics has been proposed. Radiomics refers to a process that extracts high-throughput quantitative features from radiographic images and builds predictive models relating image features to genomic patterns and clinical outcomes^4^. In the past few years, a number of radiomics models have been proposed for survival prediction^5^, distant metastasis prediction^6^, molecular characteristics classification^7^, etc. The high-throughput feature extraction is a critical task in radiomics. In previous studies, most extracted features are explicitly designed, or handcrafted^8^. These handcrafted features include tumour shape, intensity, texture and wavelet textures. Although the number of handcrafted features can reach tens of thousands, these features are shallow and low-order image features. Note that according to the radiomics hypothesis, intra-tumor imaging heterogeneity could be the expression of underlying genetic heterogeneity^9^. However, shallow and low-order features may not fully characterize image heterogeneity, therefore may limit the potential of radiomics model. On the other hand, handcrafted features are limited to the current knowledge of medical imaging. In such a case, it is necessary to assess deeper and higher-order features that may improve the predictive performance of the radiomics model.

Recently, the performance of deep learning^10^ has been intensively demonstrated in computer vision^11^. Convolutional Neural Network (CNN)^12^ is a typical artificial neural network in deep learning, which has achieved state-of-the-art performances on image and video recognition^13^ and segmentation^14^. When the data sets are large enough, the deep learning algorithms often perform better compared to traditional algorithms. However, when it comes to medical image analysis domain, the data sets are often inadequate to reach full potential of deep learning. In computer vision domain, transfer learning and fine tuning are often used to solve the problem of a small data set^15^. Transfer learning can also be incorporated into current radiomics model for extraction of a large number of deep features from hidden layers of CNN. These deep features contain more abstract information of medical images and may provide more predictive patterns compared with the handcrafted features. To the best of our knowledge, little work has been done on construction and evaluation of deep feature-based radiomics models.

**Figure 1:**
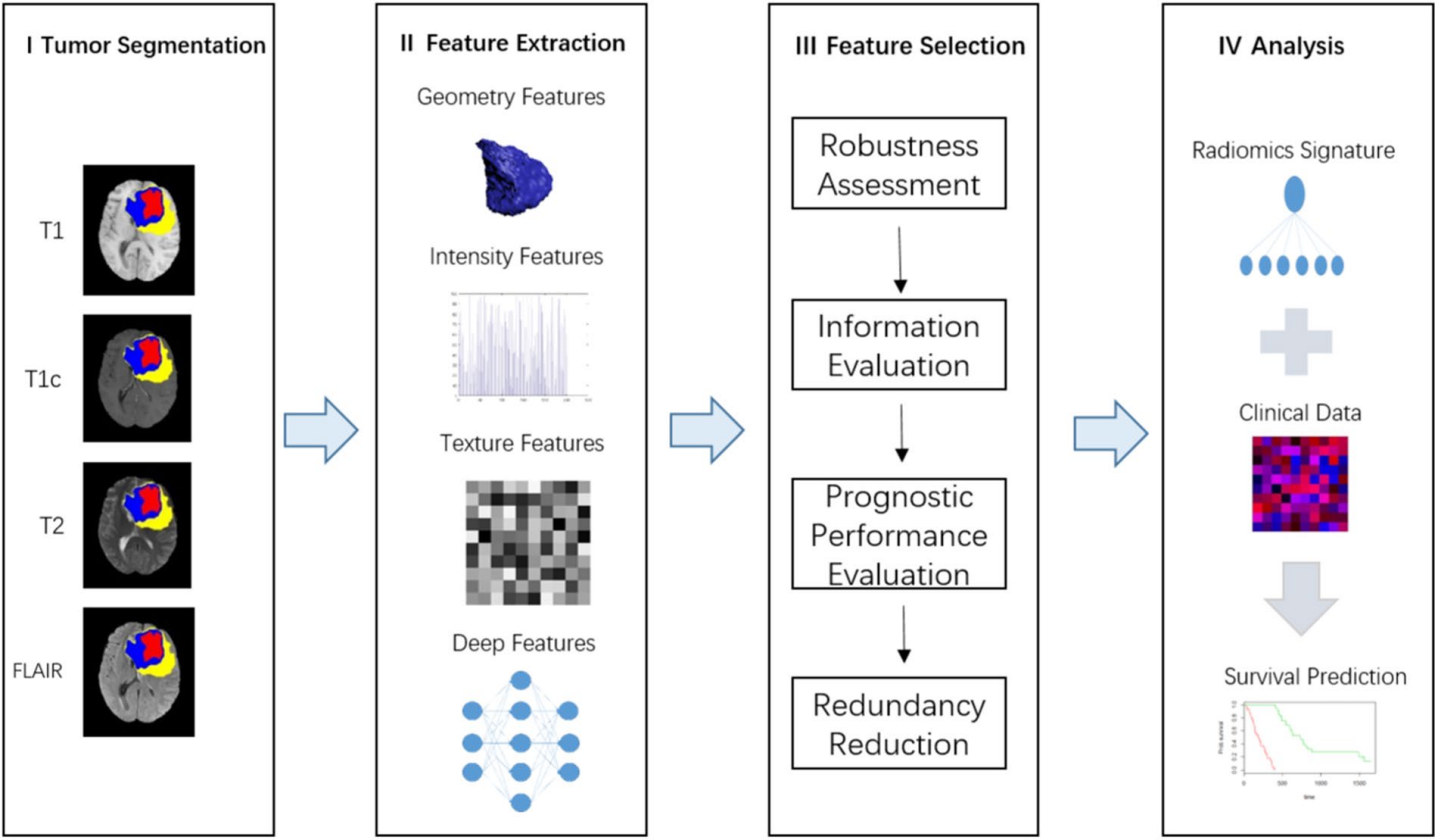
The workflow of radiomics analysis in this study.

In this work, we propose a deep feature-based radiomics model for prediction of OS in GBM patients. Both handcrafted features and deep features were extracted from multi-modality MR images. Deep features were extracted from the pre-trained CNN via transfer learning. After a four-step feature selection method, six most robust, nonredundant and predictive features were selected. Finally, a radiomics signature as well as a radiomics nomogram were constructed on a discovery cohort and validated on an independent validation cohort. Figure 1 shows the workflow of radiomics analysis in this study.

## Methods

### Data Sets

In this study, a total of 112 patients (62 men and 50 women; mean age, 54.640 years ±44.040; range 10–84 years) with pathologically confirmed GBM were included. The patient cohorts consisted of two groups: a discovery cohort comprising 75 patients from the Cancer Genome Archive (TCGA) database^16^, and an independent validation cohort comprising 37 patients collected in Sun Yat-Sen University Cancer Center, Guangzhou, China. The demographic and clinical characteristics of patients in the discovery data set and validation data set are shown in Table 1. The imaging procedure, data processing and experiment design were approved by the Sun Yat-Sen University Cancer Center Ethics Committee. All methods were carried out in accordance with relevant guidelines. As TCGA are publicly available database without patient identifier, no institutional review board approval is required for the discovery data set. For the validation data, informed consent was obtained from all subjects. The inclusion criteria were that patients with newly diagnosed and treatment-naive GBM and survival information and pre-treatment MR imaging including T1-weighted, T1-weighted Gadolinium contrast-enhanced, T2-weighted, and T2-weighted FLAIR images (short for T1, T1C, T2, and FLAIR). The exclusion criteria are patients with a history of surgery or chemoradiation therapy and patients missing survival information. Overall survival is calculated from the initial pathologic diagnosis date to death or censure point if still alive. The MRI data of the discovery cohort is obtained from the Cancer Imaging Archive (TCIA) that include imaging data corresponding to TCIA patients.

**Table 1:** Demographic and Clinical Characteristics of Patients in the Discovery Data set and Validation Data Set.

Characteristic	Discovery Data Set	Validation Data Set
No. of patients*	75 (67%)	37 (33%)
Sex^+^(*P* = 0.553)		
Male^*^	43 (57%)	32 (43%)
Female^+^	19 (51%)	18 (49%)
Age^+^(*P* = 0.909)		
Ranges	19–84	10–78
Median^†^	57 (52–59)	55 (49–62)
Mean^†^	54.990 (51.710–58.260)	53.950 (48.240–59.650)
OS^+^(*P* = 0.978)		
Ranges	30–1642	77–1870
Median^†^	441 (381–530)	377 (332–584)
Mean^†^	495.160 (412.520–577.800)	494.220 (364.250–624.180)

*Data in parentheses are percentages. ^+^Data in parentheses are P value. ^†^Data in parentheses are 95 percent confidence interval.

### Image Preprocessing and Tumor Segmentation

First, the T1, T1C, T2, and FLAIR images were preprocessed, encompassing N4 correction of bias field, skull stripping, image resampling to 1 *mm* × 1 *mm* × 1 *mm* isotropic voxels with a linear interpolater, rigid registration using T1C image as a template, and intensity normalization by histogram matching. All preprocessing were performed with the open source software ITK. Then, the three-dimensional tumor subregions were segmented manually by two neurosurgeons (Y.C. and J.Z., with 8 and 10 years of experience in neuroimaging and neurosurgical oncology, respectively). The segmentation was performed in transverse sections slice by slice with the open source software 3D Slicer version 4.5.0-1 (https://www.slicer.org/)^17^. Three tumor subregions were segmented, including the necrosis area, the enhancement area and the edema area. The necrosis area was the low intensity necrotic structures within the enhancing rim in T1C and had hyper-intense signal in T2 and FLAIR. The enhancement area was confirmed as the Gadolinium enhancing rim excluding the necrotic center and hemorrhage with both T1C and T1 images. The edema area was identified as abnormality visible in T2 and FLAIR excluding ventricles and cerebrospinal fluid. The edema area may include both peritumoral edema and any non-enhancing tumor. The segmented tumor subregions were then used for feature extraction.

### Feature Extraction

#### Handcrafted Features

The handcrafted features were extracted from five subregions and four MR modalities. The feature extraction subregions include necrosis, enhancement, edema, tumor core (the whole tumor except edema) and whole tumor (necrosis, enhancement and edema). The handcrafted features can be divided into three groups: (I) geometry, (II) intensity and (III) texture. The geometry features describe the three-dimensional shape characteristics of the tumor. The intensity features describe the first-order statistical distribution of the voxel intensities within the tumor. The texture features describe the patterns, or the second- and high-order spatial distributions of the intensities. Here the texture features are extracted using several different methods, including the gray-level co-occurrence matrix (GLCM), gray-level run length matrix (GLRLM), gray level size zone matrix (GLSZM) and neighborhood gray-tone difference matrix (NGTDM) methods^8^. A total of 1403 handcrafted features are extracted, including 23 geometry features, 340 intensity features, and 1040 texture features. Details of the handcrafted features can be found in Supplementary Table 1. All handcrafted features are extracted with an in-house feature analysis program implemented in Matlab 2010a (Mathworks, Natick, Mass).

#### Deep Features

**Figure 2:**
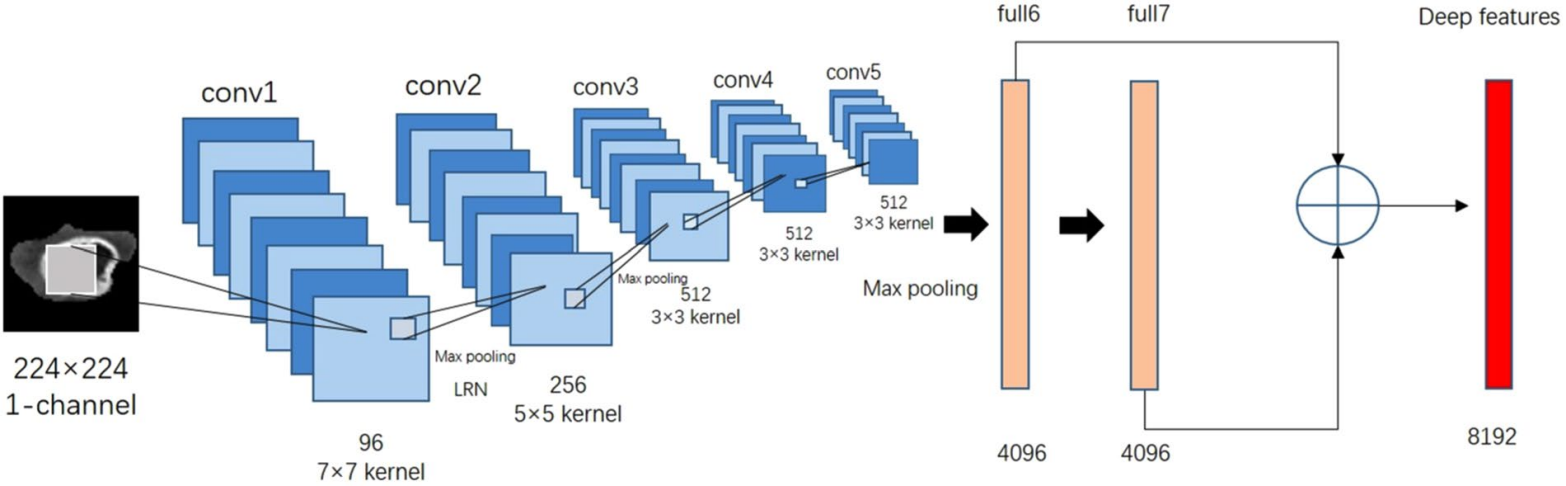
Illustration of deep features extraction. LRN is short for Local Response Normalization. The details of the CNN_S framework can be found in Supplementary Table 2.

Deep features were extracted from pre-trained CNN via transfer learning. In this study, CNN_S was chosen as the pre-trained CNN model^18^. CNN_S contained five convolution layers and three fully-connected layers. The hyper-parameters of CNN_S were weight decay 5 × 10^−4^, momentum 0.9, initial learning rate 10^−2^. When the validation error stopped diminution, initial rate was down to one tenth. The CNN_S model was trained on the ILSVRC-2012 dataset (all weights of CNN_S were predetermined), and the top-5 classification error on the validation was 13.1%. For each patient, the necrosis, tumor core and whole tumor subregions were chosen as input of CNN_S. First, from multiple transverse slices in the segmentation volume, we picked out the slice which had the largest tumor area. Then, the gray values were normalized to range [0, 255] using linear transformation. Based on the segmentation results, the three tumor subregions were cropped from the selected slices in all four MR modalities. Next, each cropped subregion image was resized to 224 × 224 with bicubic interpolation. The obtained images can be used as the model input. The G and B channels of CNN_S were turned off so only grayscale images were allowed to enter the model. Finally, the deep features can be computed by only forward propagation and were extracted from fully-connected layer 6 and fully-connected layer 7. In total, 98304 (4 × 3 × 2 × 4096) deep features can be extracted for each patients. This procedure was accomplished by using the deep learning toolkit CAFFE^19^. The deep feature extraction is shown in Fig. 2.

### Feature Selection

After features extraction, all 1403 handcrafted features and 98304 deep features for each patients were normalized as z-scores. Having these high-dimensional imaging features, a feature selection is required to avoid overfitting while improve both the generalizabiliy and interpretability of the training-based radiomics model. Here a four-step method is used for feature selection. All calculations are performed on the discovery data set.

First, the robustness of the image features was evaluated. As the feature calculation depends on the tumor subregion contours, image features that are robust against tumor segmentation uncertainties were selected. Here both test-retest analysis and inter-rater analysis were used to determine the feature robustness. Based on 30 patients randomly chosen from the discovery data set, the test-retest analysis was performed where for each patient the tumor subregions were segmented twice by one rater (Y.C.). The data set used for inter-rater analysis included another 30 randomly chosen patients, where for each patient the tumor subregions were segmented by two raters independently. The features extracted from these multiple-segmented subregions were assessed using intraclass correlation coefficient (ICC)^20^. Feature with ICC ≥ 0.85 were considered as robust against intra- and inter-rater uncertainties. After robustness evaluation, 85392 out of the initial 99707 image features remained.

Then, the median absolute deviations (MAD) was calculated for each remained feature^21^. Features with MAD equal to zero were discarded, as these features were considered as non-informative. After this step, 33881 features were left. Next, we further selected features with prognostic value. Here the prognostic performance is assessed using the concordance index (C-index), a generalization of the area under the receiver operating characteristic (ROC) curve (AUC)^22^. The C-index for each feature was calculated. Features with C-index ≥ 0.580 are considered as predictive factors. After prognostic performance analysis, 1581 features remained. Then, we further reduced the data dimension by removing highly correlated features. Here the correlation coefficient between each pair of features is calculated. For feature pair with correlated coefficient ≥0.90, the more prognostic feature is retained and the other feature is removed. Finally, the remained 150 image features are selected and regarded as robust, predictive and nonredundant.

### Statistical Analysis

#### Clinical Characteristics

The statistical analysis was performed with R software version 3.3.2 (http://www.R-project.org)^23^ and X-tile software version 3.6.1 (Yale University School of Medicine, New Haven, Conn)^24^. The differences in age, sex, tumor volume, KPS and overall survival between the discovery and the validation data sets were assessed using an independent sample *t* test, Mann-Whitney *U* test or *χ* ^2^ test, where appropriate.

#### Signature Construction

Based on the selected 150 features, we aimed to construct a radiomics signature using multivariate Cox regression model for prediction of survival in GBM patients. Because there were more image features than patients, strong feature selection and shrinkage were still required to prevent overfitting as well as increase interpretation. To address this problem, the least absolute shrinkage and selection operator (LASSO) Cox regression model was used on the discovery data set for signature construction^25^. Depending on the regulation weight *λ*, LASSO shrinks all regression coefficients towards zero and sets the coefficients of many irrelevant features exactly to zero. To find an optimal *λ*, 10-fold cross validation with minimum criteria was employed, where the final value of *λ* yielded minimum cross validation error. The retained features with nonzero coefficients were used for regression model fitting and combined into a radiomics signature. Subsequently, we obtained a radiomics score for each patient by a linear combination of retained features weighed by their model coefficients. The R package glmnet was used for LASSO Cox regression modeling.

#### Signature Validation

The association of the constructed signature with survival was assessed on the discovery data set and validated on the validation data set by using Kaplan-Mier survival analysis. Based on a threshold calculated using the radiomics score, all patients were stratified into high-risk and low-risk groups. The threshold was estimated based on the discovery data set by using an optimal cutpoint analysis with X-tile software, and tested on the validation data set. A weighted log-rank test (G-rho rank test, rho = 1) was used to test the significant difference between the high-risk and low-risk groups. The C-Index was used to assess the performance of the signature.

To assess the univariate predictive performance of each feature with non-zero LASSO coefficient, the univariate analysis was performed based on both discovery and validation data sets. To assess the univariate association with OS, each non-zero feature was used for patient stratification into high-risk and low-risk groups.

To compare the built radiomics signature with other clinical risk factors such as age and KPS, the C-indices of these clinical risk factors were calculated based on both discovery and validation data sets. To assess the combinative prognostic value of the signature with clinical factors, we put the radiomics signature together with clinical parameters into the Cox regression model. The model was fitted based on the discovery data set and validated on the validation data set. The R package survcomp was used for the survival analysis.

#### Radiomics Nomogram

Furthermore, to intuitively and efficiently assess the incremental prognostic value of the radiomics signature to the clinical risk factors (age and KPS), a radiomics nomogram was presented on the validation data set. The nomogram combined the radiomics signature and the clinical risk factors based on the multivariate Cox analysis. To compare the agreement between the OS prediction of the nomogram and the actual observation, the calibration curve was calculated.

## Results

### Clinical Characteristics and OS

The median and mean of OS were 441 days and 495.160 days for the discovery set, 377 days and 494.220 days for the validation set. The median and mean of age were 57 years and 54.990 years for the discovery set, 55 years and 53.950 years for the validation set. The discovery set had 43 males and 32 females, while the validation set had 19 males and 18 females. There was no significant difference in clinical and follow-up data between the discovery and validation data sets (*P* = 0.553 for sex test, 0.748 for KPS test, 0.909 for age test, 0.302 for tumor volume test and 0.978 for OS test).

### Signature Construction

There were six features with non-zero coefficients in the LASSO Cox regression model: FLAIR_ST_F7_870, FLAIR_SN_F7_2297, T1C_SNE_F6_806, T2_SNE_F7_772, T1C_SNE_F7_1508 and FLAIR_SNE_F6_2981. Introduction of the six features can be seen in Supplementary Table 3. For example, T1C_SNE_F7_1508 indicated that this feature was extracted from tumor core in T1C and was taken from the 1508th neurons of the fully-connected layer 7. The optimal *λ* selection in LASSO Cox regression model is shown in Supplementary Figure 1. By linearly combining the six features, the radiomics signature can be constructed, and the radiomics score can be computed as

$$\begin{array}{rcl}Radiomics\_signature\_score & = & FLAIR\_ST\_F\mathrm{7\_870}\times 0.06867720\\  &  & +FLAIR\_SN\_F\mathrm{7\_2297}\times (-\mathrm{0.05909112)}\\  &  & +T1C\_SNE\_F\mathrm{6\_806}\times 0.05420293\\  &  & +T\mathrm{2\_}SNE\_F\mathrm{7\_772}\times (-\mathrm{0.03454031)}\\  &  & +T1C\_SNE\_F\mathrm{7\_1508}\times (-\mathrm{0.02240571)}\\  &  & +FLAIR\_SNE\_F\mathrm{6\_2981}\times (-\mathrm{0.00958802)}\end{array}$$

### Signature Validation

**Figure 3:**
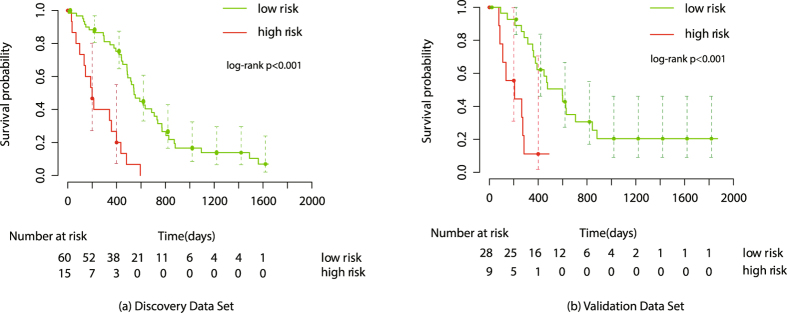
Illustration of Kaplan-Meier survival curve. The Kaplan-Meier survival curve show OS risk stratification for patients in Discovery data set (**a**) and Validation data set (**b**). Patients were classified as low risk and high risk according to radimics signature. The vertical dashed line is 95% confidence interval.

The radiomics signature achieved a C-Index of 0.731 (95% confidence intervals [CI]: 0.645, 0.817) for the discovery data set, and 0.710 (95% CI: 0.588, 0.932) for the independent validation data set, demonstrating the predictive performance of the model. Based on the radiomics score of patients in the discovery data set, the optimal cutoff calculated by the X-tile plot was 0.1343235, as shown in Supplementary Figure 2. Then, patients in both the discovery and validation data sets were stratified into low-risk (Rad-score < 0.1343235) and high-risk (Rad-score>0.1343235) groups, as shown in Fig. 3. The significant association of the radiomics signature with OS was shown in discovery data set (P < 0.001, hazard ratio [HR] = 5.042, 95% CI: 2.624, 9.689), and confirmed in the validation data set (P < 0.001, HR = 5.128, 95% CI: 2.029, 12.960). The OS in the low-risk and high-risk groups in the discovery and validation data sets are listed in Supplementary Table 4.

**Figure 4:**
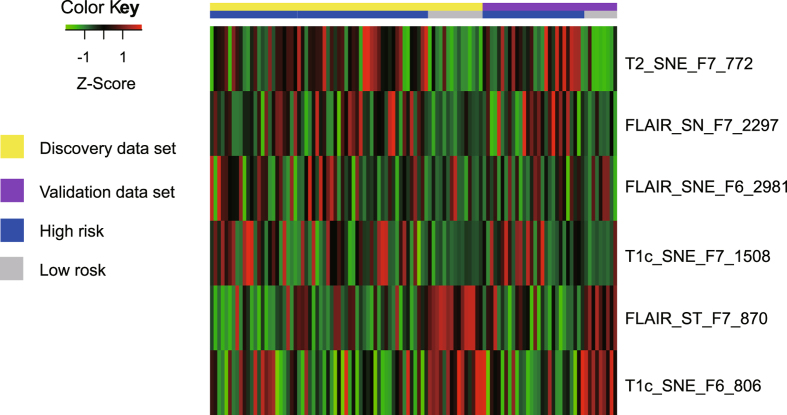
The heat map of selected radiomics feature. Each row of the heat map represents a radiomics feature and each column represents a patient. The Z-Score difference of each radiomics feature between high risk and low risk group can be seen from the heat map. At the same time, it can be observed directly from the heat map that there is a consistency of radiomics feature Z-Score between the discovery data set and the validation data set.

The heatmap of the six radiomics features is shown in Fig. 4. The univariate analysis results based on the validation data set are shown in Table 2. The univariate Kaplan-Meier survival curves for each feature can be found in Supplementary Figure 3. It shows that two individual features succeeded to stratify patients in the validation data set into high-risk and low-risk groups, with G-rho rank test P value of 0.003 for FLAIR_ST_F7_870, and <0.001 for T1C_SNE_F6_806.

**Table 2:** Univariate prognostic value of non-zero deep features in the validation data set.

Feature	C-index (95% CI)	*P* Value	Hazard Ratio (95% CI)
FLAIR_ST_F7_870	0.680 (0.562, 0.799)	0.003	4.980 (0.562, 19.870)
FLAIR_SN_F7_2297	0.620 (0.502, 0.738)	0.230	1.572 (0.746, 3.311)
T1C_SNE_F6_806	0.648 (0.526, 0.770)	<0.001	6.785 (2.126, 21.660)
T2_SNE_F7_772	0.616 (0.494, 0.738)	0.109	1.953 (0.849, 4.493)
T1C_SNE_F7_1508	0.609 (0.493, 0.725)	0.452	1.359 (0.609, 3.034)
FLAIR_SNE_F6_2981	0.554 (0.434, 0.675)	0.452	1.331 (0.630, 2.811)

The C-indices of the clinical parameters were 0.621 (95% CI: 0.499, 0.743) for age and 0.549 (95% CI: 0.432, 0.666) for KPS in the validation data set. None of them successfully stratified the patients into high-risk and low-risk groups in the validation data set, with G-rho rank test P value of 0.324 for age and 0.620 for KPS. In the validation data set, the combined Cox model was demonstrated to be associated with the OS (C-index: 0.739 [95% CI: 0.686, 0.792], HR: 4.608 [95% CI: 1.884, 11.270]) and to stratify the patients into high-risk and low-risk groups with G-rho test P value < 0.001.

### Radiomics Nomogram

**Figure 5:**
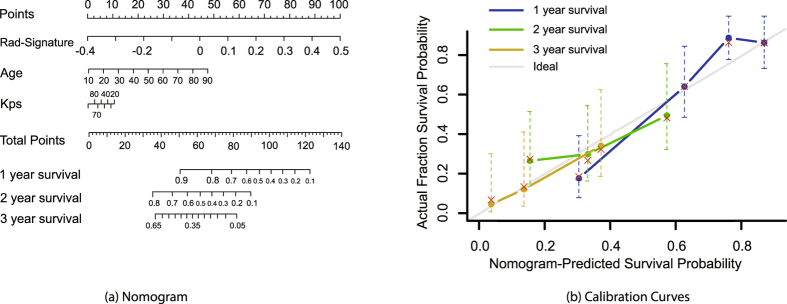
The nomogram (**a**) and calibration (**b**) curves. Radiomics signature and clinical data are associated with survival probability of 1, 2 and 3 years. The predictors are radiomics signature score, age of the patient (in years) and Karnofsky performance score (KPS). Draw a vertical line from each predictor to ‘Points’ to get the score of the predictor. Then summing up the scores of each predictor, the ‘Total Points’ correspond to the survival probability of 1, 2 and 3 years by drawing a vertical line from ‘Total Points’ to each survival probability axis. Calibration curves is used to assess the consistency between nomogram-predicted survival probability and actual fraction survival probability.

The radiomics nomogram and corresponding calibration curve were shown in Fig. 5. It shows intuitively that the proposed radiomics nomogram performed better than age and KPS on survival prediction in patients with GBM.

## Discussion

In this paper we present a prognostic radiomics model that feature extraction is no longer limited to handcrafted features. Higher-order deep features were extracted and incorporated into our radiomics model. The CNN_S model which pre-trained from natural image dataset was used as deep feature extractor. Based on multi-modality MR images (T1, T1C, T2 and T2 FLAIR), high-throughput handcrafted and deep features were extracted. After a four-step feature selection, a six-feature radiomics signature was constructed using LASSO Cox model for prediction of overall survival in patients with GBM. The radiomics signature was demonstrated to be associated with the OS and successfully stratified patients into high-risk and low-risk groups. We can further improve the prediction performance by combining the radiomics signature with established clinical risk factors such as age and KPS. By combining radiomics signature with clinical factors into a radiomics nomogram, the OS of GBM patients can be effectively predicted.

The radiomics signature consisted of six imaging features: FLAIR_ST_F7_870, FLAIR_SN_F7_2297, T1C_SNE_F6_806, T2_SNE_F7_772, T1C_SNE_F7_1508 and FLAIR_SNE_F6_2981. It can be seen that the six selected features were all deep features, extracted from multiple tumor subregions in T1C, T2 and FLAIR images. It is demonstrated that the deep features extracted via transfer learning performed better than traditional handcrafted features in prediction of OS in GBM patients. The result is not surprising, as deep features reflect higher order imaging patterns and capture more imaging heterogeneity compared with low-level shape, intensity and texture features. According to the radiomics hypothesis, intra-tumor imaging heterogeneity could be the expression of underlying genetic heterogeneity, which could develop resistances to treatment and thusly indicate poorer prognosis. However, the interpretation of the association between the deep features and the genetic characteristics remains challenging. It is related to complex biological process. Future work is needed to establish a radiogenomics rationale to explain the correlation between deep imaging features and genetic heterogeneity.

Two individual features achieved log-rank P values of 0.003 and P value <0.001 respectively in the validation data set. The other four individual features failed to stratify patients into high-risk and low-risk groups in the validation data set. The multi-feature signature was successful to predict the OS of patients in the validation data set and performed better than any individual feature. From the statistical perspective, nonsignificant association with survival does not mean less importance. On the other hand, multivariate model is statistically robust in survival analysis^26^. Moreover, the intra-tumor genetic heterogeneity suggests that tumor subregions could be genetically different and may comprise multiple subclones. This could be better reflected by multiple high-order deep features extracted from multi-subregions in multi-modalities rather than individual feature. Similar to the genomic studies of exploring biomarkers from high-throughput genomic data, it is also regarded as a common“-omics” approach to construct a multi-factor radiomics signature for outcome prediction.

In our study the proposed radiomics signature performed better than traditional risk factors such as age and KPS. None of these clinical factors successfully stratified patients into groups with different prognostic risks. After combining the radiomics signature with clinical factors into a Cox regression model, the predictive power improved with C-index of 0.739 in validation data set. According to the radiomics signature and the two clinical risk factors, we drew a nomogram that can visually predict the probability of survival. According to the calibration curve we can see that our nomogram had good predictive performance.

Despite the promising results, this study still has several limitations. First, this is a retrospective study with relatively small sample size, although independent validation cohort from local institution was used. In future, large-scale multicenter study is required to fully assess the generalization ability of the radiomics model. Second, due to the limitation of the small sample size, this study employed transfer learning for extraction of deep features. Further work is needed to train an dedicated feature extractor by fine-tuning on a pre-trained network or training from scratch. A deep feature extractor that explicitly designed for MRI GBM radiomics model should be established. Third, the association between deep features and underlying genetic characteristics was not investigated. In future more work should be done to explore the potential radiomics-genomics correlation in GBM patients.

In conclusion, we have proposed a six-deep-feature radiomics signature that have the potential to be an imaging biomarker for prediction of the OS in patients with GBM. It was demonstrated that the deep learning method can be incorporated into the state-of-the-art radiomics model to achieve a better performance. The proposed signature predicted the OS in GBM patients with better performance compared with conventional factors such as age and KPS. A nomogram was proposed for prediction of the probability of survival. Despite the limitations, the proposed radiomics model has the potential to facilitate the preoperative care of patients with GBM.

## Electronic supplementary material

Supplementary Information

## References

[1] Bleeker FE, Molenaar RJ, Leenstra S (2012). Recent advances in the molecular understanding of glioblastoma. Journal of neuro-oncology.

[2] Young, R. M., Jamshidi, A., Davis, G. & Sherman, J. H. Current trends in the surgical management and treatment of adult glioblastoma. *Annals of translational medicine* **3** (2015). 10.3978/j.issn.2305-5839.2015.05.10 PMC4481356 26207249

[3] Dolecek TA, Propp JM, Stroup NE, Kruchko C (2012). Cbtrus statistical report: primary brain and central nervous system tumors diagnosed in the united states in 2005–2009. Neuro-oncology.

[4] Lambin P (2012). Radiomics: extracting more information from medical images using advanced feature analysis. European journal of cancer.

[5] Huang Y (2016). Radiomics signature: A potential biomarker for the prediction of disease-free survival in early-stage (i or ii) non—small cell lung cancer. Radiology.

[6] Coroller TP (2015). Ct-based radiomic signature predicts distant metastasis in lung adenocarcinoma. Radiotherapy and Oncology.

[7] Kickingereder P (2016). Radiogenomics of glioblastoma: Machine learning–based classification of molecular characteristics by using multiparametric and multiregional mr imaging features. Radiology.

[8] Aerts, H. J. *et al*. Decoding tumour phenotype by noninvasive imaging using a quantitative radiomics approach. *Nature communications* **5** (2014). 10.1038/ncomms5006 PMC4059926 24892406

[9] Gillies RJ, Kinahan PE, Hricak H (2015). Radiomics: images are more than pictures, they are data. Radiology.

[10] Hinton GE, Salakhutdinov RR (2006). Reducing the dimensionality of data with neural networks. science.

[11] Karpathy, A. *et al*. Large-scale video classification with convolutional neural networks. In *Proceedings of the IEEE conference on Computer Vision and Pattern Recognition*, 1725–1732 (2014).

[12] LeCun Y, Bottou L, Bengio Y, Haffner P (1998). Gradient-based learning applied to document recognition. Proceedings of the IEEE.

[13] He, K., Zhang, X., Ren, S. & Sun, J. Deep residual learning for image recognition. In *Proceedings of the IEEE Conference on Computer Vision and Pattern Recognition*, 770–778 (2016).

[14] Chen, L.-C., Papandreou, G., Kokkinos, I., Murphy, K. & Yuille, A. L. Semantic image segmentation with deep convolutional nets and fully connected crfs. *arXiv preprint arXiv:1412*.7062 (2014). 10.1109/TPAMI.2017.2699184 28463186

[15] Pan SJ, Yang Q (2010). A survey on transfer learning. IEEE Transactions on knowledge and data engineering.

[16] Weinstein JN (2013). The cancer genome atlas pan-cancer analysis project. Nature genetics.

[17] Fedorov A (2012). 3d slicer as an image computing platform for the quantitative imaging network. Magnetic resonance imaging.

[18] Chatfield, K., Simonyan, K., Vedaldi, A. & Zisserman, A. Return of the devil in the details: Delving deep into convolutional nets. *arXiv preprint arXiv:1405*.3531 (2014).

[19] Jia, Y. *et al*. Caffe: Convolutional architecture for fast feature embedding. In *Proceedings of the 22nd ACM international conference on Multimedia*, 675–678 (ACM, 2014).

[20] Fleiss JL, Cohen J (1973). The equivalence of weighted kappa and the intraclass correlation coefficient as measures of reliability. Educational and psychological measurement.

[21] Pham-Gia T, Hung T (2001). The mean and median absolute deviations. Mathematical and Computer Modelling.

[22] Pencina MJ, D’Agostino RB (2004). Overall c as a measure of discrimination in survival analysis: model specific population value and confidence interval estimation. Statistics in medicine.

[23] Team, R. C. R: A language and environment for statistical computing. r foundation for statistical computing, vienna, austria. 2013 (2014).

[24] Camp RL, Dolledfilhart M, Rimm DL (2004). X-tile a new bio-informatics tool for biomarker assessment and outcome-based cut-point optimization. Clinical Cancer Research An Official Journal of the American Association for Cancer Research.

[25] Tibshirani R (1997). The lasso method for variable selection in the cox model. Statistics in medicine.

[26] Moons KG (2015). Transparent reporting of a multivariable prediction model for individual prognosis or diagnosis (tripod): Explanation and elaborationthe tripod statement: Explanation and elaboration. Annals of internal medicine.

