# Supplementary material for: A Deep Learning-Based Radiomics Model for Prediction of Survival in Glioblastoma Multiforme
Source: Sci Rep. 2017 Sep 4;7:10353. doi: 10.1038/s41598-017-10649-8 (PMC5583361; doi:10.1038/s41598-017-10649-8)
Supplement: Supplementary file 1 — Supplementary Information [file 41598_2017_10649_MOESM1_ESM.pdf]

Supplementary Information
A Deep Learning-Based Radiomics Model for Prediction of Survival in Glioblastoma Multiforme

Jiangwei Lao[1,+], Yinsheng Chen[2,+], Qihua Li[3], Ji Zhang[2], Jing Liu[4], Zhi-Cheng Li[3,*], and Guangtao Zhai[1,*]

[1]Institute of Image Communication and Network Engineering, Shanghai Jiao Tong University, Shanghai, China
[2]Department of Neurosurgery/Neuro-oncology, Sun Yat-sen University Cancer Center, State Key Laboratory of Oncology inSouth China, Collaborative Innovation Center for Cancer Medicine, Guangzhou, China.
[3]Institute of Biomedical and Health Engineering, Shenzhen Institutes of Advanced Technology, Chinese Academy of Sciences, Shenzhen, China.
[4]School of Electrical and Information Engineering, Tianjin University, Tianjin, China.
[*]Corresponding authors: zc.li@siat.ac.cn, zhaiguangtao@sjtu.edu.cn
[+]These authors contributed equally to this work

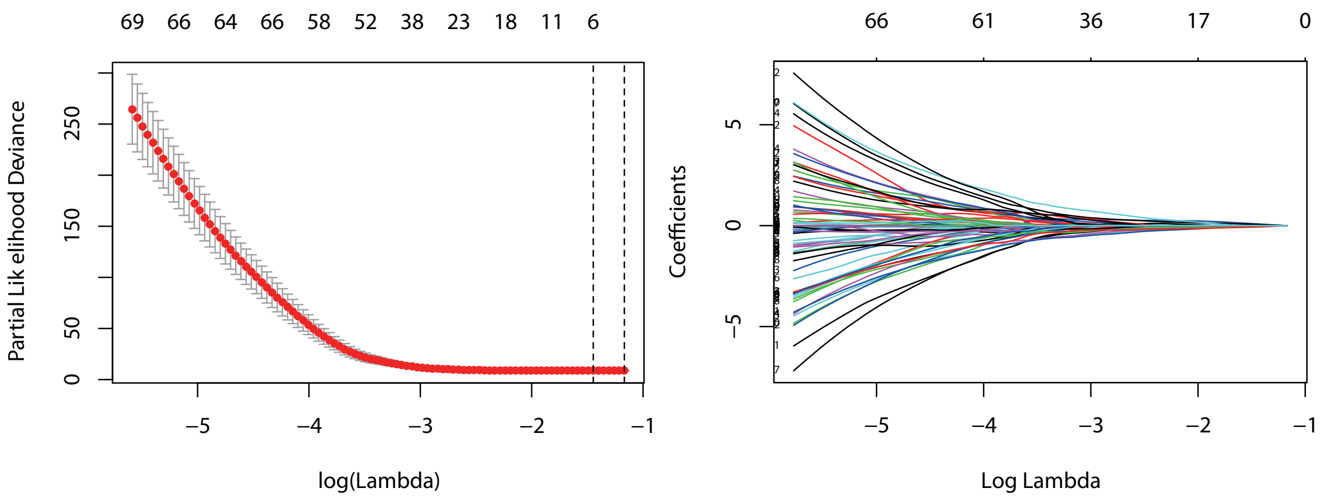

(a) (b)

Supplementary Figure 1: Illustration of feature selection using the LASSO Cox regression. (a) The left vertical line in our plot shows us where the CV-error curve hits its minimum. The right vertical line shows us the most regularized model with CV-error within 1 standard deviation of the minimum. A value λ of 0.2352557 was chosen according to 10-fold cross validation. (b) LASSO coefficient profiles of the 150 features.

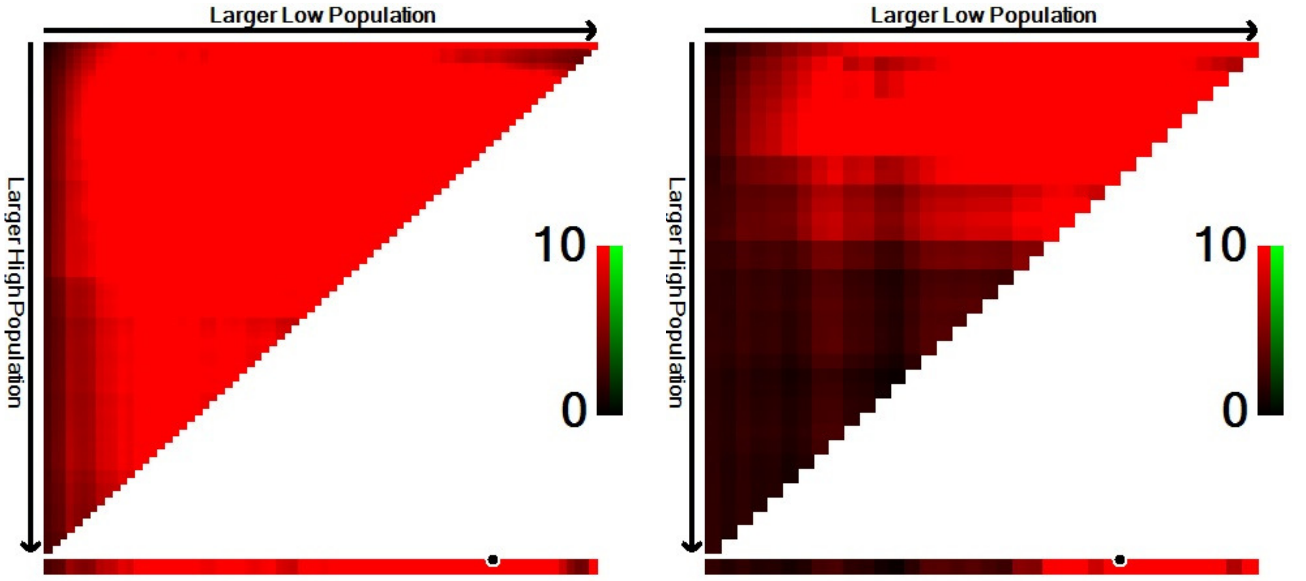

(a) Disocovery Data set

(b) Validation Data Set

Supplementary Figure 2: Illustration of X-tile plot of the radiomics signature. The colors in the plot represent the strength of the association at each division, ranging from low (black) to high (bright red or green). Red represents the inverse association between the Rad-score and survival. The x-axis represents all potential cutoff points, from low to high (left to right), that define a low subset, whereas the y-axis represents cutoff points from high to low (top to bottom) that define a high subset. The optimum cut point is highlighted by the black dot on the x-axis.

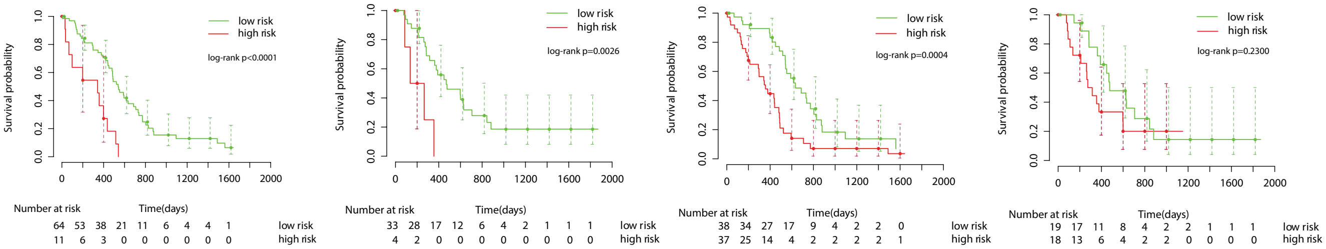

(a) FLAIR_ST_F7_870 Discovery **(b)** FLAIR_ST_F7_870 Validation **(c)** FLAIR_SN_F7_2297 Discovery **(d)** FLAIR_SN_F7_2297 Validation

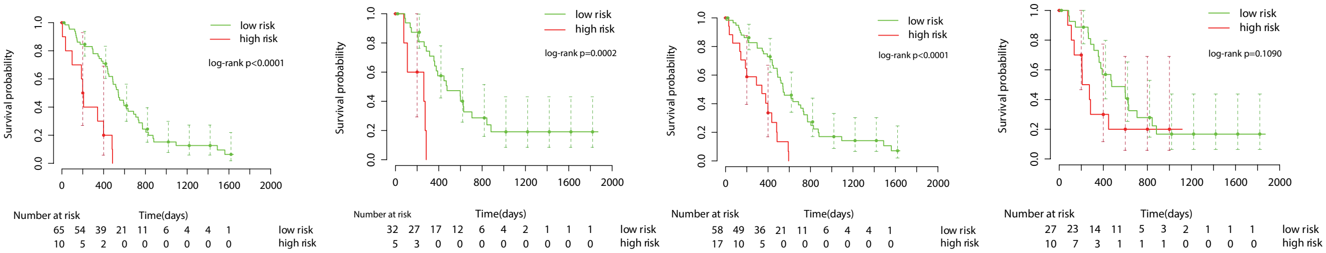

(e) T1c_SNE_F6_806 Discovery **(f)** T1c_SNE_F6_806 Validation **(g)** T2_SNE_F7_772 Discovery **(h)** T2_SNE_F7_772 Validation

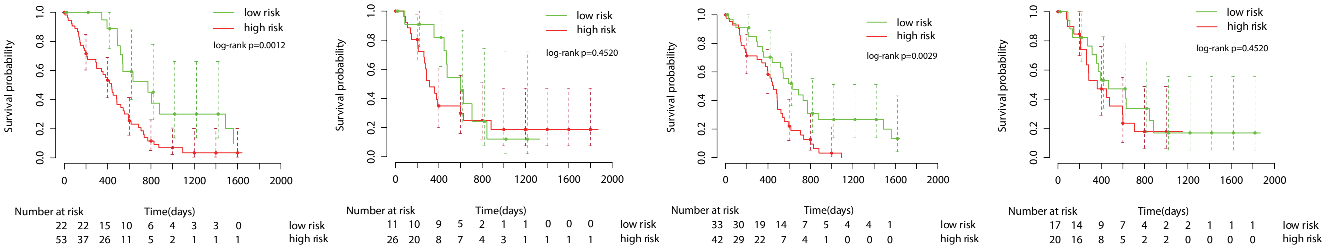

(i) T1c_SNE_F7_1508 Discovery **(j)** T1c_SNE_F7_1508 Validation **(k)** FLAIR_SNE_F6_2981 Discovery **(l)** FLAIR_SNE_F6_2981 Validation

Supplementary Figure 3: Kaplan-Meier survival curve based on single radiomics feature prediction. In the discovery data set, the hazard ratio (HR) of single radiomics feature prediction is 3.509 (3.807 (FLAIR_ST_F7_870, 95% CI: 1.883, 7.697), 2.545 (FLAIR_SN_F7_2297, 95% CI: 1.5, 4.318), 4.777 (T1c_SNE_F6_806, 95% CI: 2.251, 10.14), 3.509 (T2_SNE_F7_772, 95% CI: 1.867, 6.595), 2.712 (T1c_SNE_F7_1508, 95% CI: 1.448, 5.082), 2.285 (FLAIR_SNE_F6_2981, 95% CI: 1.309, 3.991). In the validation data set, the hazard ratio (HR) of single radiomics feature prediction is 4.98 (FLAIR_ST_F7_870, 95% CI: 1.562, 15.87), 1.572 (FLAIR_SN_F7_2297, 95% CI: 0.7463, 3.311), 6.785 (T1c_SNE_F6_806, 95% CI: 2.126, 21.66), 1.953 (T2_SNE_F7_772, 95% CI: 0.849, 4.493), 1.359 (T1c_SNE_F7_1508, 95% CI: 0.6092, 3.034), 1.331 (FLAIR_SNE_F6_2981, 95% CI: 0.6304, 2.811).

Geometry Features	Volume, Surface Area*, Longest Diameter*, Solidity*, Eccentricity*, Compactness1*, Compactness2*, Spherical Disproportion*, Sphericity*, Ratio of Surface Area to Volume*.
Intensity Features<br>Gray Value	Maxvalue, Medianvalue, Minvalue, Meanvalue, Energy, Entropy, Variance, Kurtosis, Root Mean Square, Skewness, Meanvalue+, Standard Deviation, Energy, Variance+, Root Mean Square+, Standard Deviation+, Mean Absolute Deviation+.
Second-order<br>Texture Features<br>GLCM	Contrast, Correlation, Difference Entropy, Entropy, Informational Measure of Correlation 1 (IMC1), Sum Average, Sum Entropy, Informational Measure of Correlation 2 (IMC2), Sum Variance, Variance, Difference Variance, Autocorrelation, Dissimilarity, Inverse Difference Moment Normalized (IDMN), Inverse Difference Normalized (IDN), Cluster Prominence, Cluster Shade, Energy, Homogeneity 1, Homogeneity 2, Maximum Probability.
High-order<br>Texture Features<br>GLRLM	Short Run Emphasis (SRE), Long Run Emphsis (LRE), Gray-Level Non-uniformity (GLN), Run-Length Non-uniformity (RLN), Run Percentage (RP), Low Gray-Level Run Emphasis (LGRE), High Gray-Level Run Emphasis (HGRE), Short Run Low Gray-Level Emphasis (SRLGE), Short Run High Gray-Level Emphasis (SRHGE), Long Run Low Gray-Level Emphasis (LRLGE), Long Run High Gray-Level Emphasis (LRHGE), Gray-Level Variance (GLV), Run-Length Variance (RLV).
High-order<br>Texture Features<br>GLSZM	Small Zone Emphasis (SZE), Large Zone Emphasis (LZE), Gray-Level Non-uniformity (GLN), Zone-Size Non-uniformity (ZSN), Zone Percentage (ZP), Low Gray-Level Zone Emphasis (LGZE), High Gray-Level Zone Emphasis (LGRE), Small Zone Low Gray-Level Emphasis (SZLGE), Small Zone High Gray-Level Emphasis (SZHGE), Large Zone Low Gray-Level Emphasis (LZLGE), Large Zone High Gray-Level Emphasis (LZHGE), Gray-Level Variance (GLV), Zone-Size Variance (RLV).
High-order<br>Texture Features<br>GLSZM	Small Zone Emphasis (SZE), Large Zone Emphasis (LZE), Gray-Level Non-uniformity (GLN), Zone-Size Non-uniformity (ZSN), Zone Percentage (ZP), Low Gray-Level Zone Emphasis (LGZE), High Gray-Level Zone Emphasis (LGRE), Small Zone Low Gray-Level Emphasis (SZLGE), Small Zone High Gray-Level Emphasis (SZHGE), Large Zone Low Gray-Level Emphasis (LZLGE), Large Zone High Gray-Level Emphasis (LZHGE), Gray-Level Variance (GLV), Zone-Size Variance (RLV).
High-order<br>Texture Features<br>NGTDM	Coarseness, Contrast, Busyness, Complexity, Strength.

Supplementary Table 1: Summary of Handcrafted Features in This Study. Unless otherwise noted, features are extracted from necrosis, enhancement, edema, tumor core (the whole tumor except edema) and whole tumor (necrosis, enhancement and edema). These features are extracted from original ROI image. *Features are extracted from tumor core and whole tumor. +Features are extracted from processed ROI image whose gray histogram is quantized to 1-100. The algorithm for radiomics features calculation can be found in [1].

Architecture	CNN_S
conv1	$96 \times 7 \times 7$<br>st. 2,pad 0<br>LRN,$\times 3$ pool
conv2	$256 \times 5 \times 5$<br>st. 1,pad 1<br>LRN,$\times 2$ pool
conv3	$512 \times 3 \times 3$<br>st. 1,pad 1
conv4	$512 \times 3 \times 3$<br>st. 1,pad 1
conv5	$512 \times 3 \times 3$<br>st. 1,pad 1<br>$\times 3$ pool
full6	4096<br>dropout
full7	4096<br>dropout
full8	1000<br>softmax

Supplementary Table 2: CNN_S architectures.

FLAIR_ST_F7_870	This feature was extracted from whole tumor in FLAIR and was taken from the 870th neurons of the fully-connected layer 7.
FLAIR_SN_F7_2297	This feature was extracted from necrosis in FLAIR and was taken from the 2297th neurons of the fully-connected layer 7.
T1C_SNE_F6_806	This feature was extracted from tumor core in T1C and was taken from the 806th neurons of the fully-connected layer 6.
T2_SNE_F7_772	This feature was extracted from tumor core in T2 and was taken from the 772th neurons of the fully-connected layer 7.
T1C_SNE_F7_1508	This feature was extracted from tumor core in T1C and was taken from the 1508th neurons of the fully-connected layer 7.
FLAIR_SNE_F6_2981	This feature was extracted from tumor core in FLAIR and was taken from the 2981th neurons of the fully-connected layer 6.

Supplementary Table 3: The details of the six features.

	Discovery Data Set			Validation Data Set		
	High-Risk Group	Low-Risk Group	Total	High-Risk Group	Low-Risk Group	Total
Number of patients	15	60	75	9	28	37
The median of OS*	199 (121.5-369)	489.5 (434.5-604.5)	441 (381-530)	206 (93.5-376.5)	472.5 (401.5-703)	377 (332-584)
Number of survival						
1 year survival	4	41	45	1	18	19
2 year survival	0	16	16	0	6	6
3 year survival	0	5	5	0	4	4

Supplementary Table 4: The OS in the low-risk and high-risk groups in the discovery and validation data sets.*Data in parentheses are 95 percent confidence interval.

References

[1] Aerts, H. J. *et al.* Decoding tumour phenotype by noninvasive imaging using a quantitative radiomics approach. *Nat. communications* **5** (2014).
